# Quantitative CT and pulmonary function in children with post-infectious bronchiolitis obliterans

**DOI:** 10.1371/journal.pone.0214647

**Published:** 2019-04-01

**Authors:** Jonghyeon Kim, Myung-Joon Kim, In Suk Sol, Myung Hyun Sohn, Haesung Yoon, Hyun Joo Shin, Kyung Won Kim, Mi-Jung Lee

**Affiliations:** 1 Department of Radiology, Severance Hospital, Research Institute of Radiological Science, Yonsei University College of Medicine, Seoul, South Korea; 2 Department of Pediatrics, Institute of Allergy, Brain Korea 21 PLUS Project for Medical Science, Severance Children’s Hospital, Yonsei University College of Medicine, Seoul, South Korea; University of Texas MD Anderson Cancer Center, UNITED STATES

## Abstract

**Objective:**

To investigate the feasibility of CT-based quantitative airway and air-trapping measurements and to assess their correlation with pulmonary function in children with post-infectious bronchiolitis obliterans (PIBO).

**Materials and methods:**

This retrospective study approved by the institutional review board included chest CT scans and pulmonary function tests (PFT) completed between January 2005 and December 2016 in children diagnosed with PIBO. The quantitative analysis of segmental and subsegmental bronchi was performed on each chest CT scan, measuring the areas or diameters of lumens, walls, or the entire airway. The air-trapping volume (ATV), the volume of lung area exhibiting lower attenuation than the mean attenuation of normal and air-trapping areas, was also measured in each lobe. Comparison analyses between CT parameters and PFT results were performed with Pearson or Spearman correlation.

**Results:**

In total, 23 patients were enrolled (mean age 7.0 ± 3.3 years; range, 4–15 years). We successfully measured 89.6% of all segmental bronchi. In the airway analysis, wall area showed a negative correlation with forced expiratory volume in one second (FEV_1_) in the majority of the pulmonary lobes. Air-trapping analyses demonstrated that ATV was negatively correlated with FEV_1_ and positively correlated with reactance at 5 Hz.

**Conclusion:**

Quantitative airway and air-trapping measurements from chest CT are feasible and correlate with pulmonary function in pediatric PIBO patients.

## Introduction

Post-infectious bronchiolitis obliterans (PIBO) is a rare but potentially irreversible and highly morbid chronic obstructive lung disease [1]. It is more common in children after a severe lower respiratory tract viral infection, especially adenovirus infection [2]. Other respiratory viruses such as respiratory syncytial virus and parainfluenza and other etiologies including *Mycoplasma pneumoniae* and measles virus have been associated with PIBO as well as cytomegalovirus infection in lung transplantations. Previous reports suggest that there would be a different prevalence of this disease in various areas of the world [2]. Histopathological features of PIBO include the concentric narrowing and obliteration of small airways due to an inflammatory process surrounding the bronchiolar lumen [3].

The signs, symptoms, and prognosis of PIBO vary. The findings of physical examination are not specific and PIBO must be suspected in any patient with bronchiolitis not improving after 3 weeks. Confirmative diagnosis of PIBO requires histopathological examination, but that is invasive and challenging due to clinical instability of the patient and the heterogeneous distribution of lesions. Therefore, in actual practice, the diagnosis is often made based on clinical features and computed tomography (CT) findings. The severity of disease and its clinical course are monitored by the pulmonary function test (PFT) [1]. However, the reliability of PFT varies depending on patient compliance, and sometimes it is difficult to assess the pulmonary function of young patients [4].

In comparison, CT is easier to perform in young patients because it depends less on patient cooperation than PFT. Although conventional CT cannot directly delineate small airways, it does reveal secondary findings that are caused by obstruction of small airways. The characteristic CT features of PIBO include a mosaic air-trapping pattern, bronchiectasis, and atelectasis with heterogeneous distribution throughout the entire lung [1,5]. The greatest risk of using CT scans with pediatric patients is radiation exposure. However, it remains the fastest and easiest method for evaluating the whole lung, and a low-radiation CT protocol has recently been developed that produces acceptable image quality using iterative reconstruction [6].

Quantitative imaging analysis is a promising application of CT for evaluating small airway disease by directly measuring the airway and indirectly quantifying the degree of air-trapping. Several studies of adult chronic obstructive pulmonary disease (COPD) and asthma have shown that quantitative CT measurements are significantly correlated with PFT results [7–10] and also meaningful in emphysema cohort study and COPDGene study [11,12]. However, only a few studies have used semi-quantitative measurements of CT findings [5,13] or quantitatively assessed the degree of air-trapping based on CT scans [14] performed in children. Moreover, no dedicated studies have applied quantitative airway analysis to CT data from pediatric patients. Recently, technical improvements have made quantitative CT airway measurement in children available in spite of low spatial resolution. Therefore, we investigated the feasibility of CT-based quantitative airway and air-trapping measurements and their correlation with PFT results in children diagnosed with PIBO.

## Material and methods

### Patients

This retrospective study was approved by the institutional review board of Severance Hospital, and the requirement for written informed consent was waived. We searched our institutional database for patients younger than 15 years who were clinically diagnosed with PIBO and underwent both chest CT scan and PFT within a one-month interval at some point between January 2005 and December 2016. Asthma was confirmed on the basis of consistent respiratory symptoms verified by physicians, the presence of either a bronchodilator response of ≥12% increase in the forced expiratory volume in one second (FEV_1_), or bronchial hyperresponsiveness defined as a decrease in FEV_1_ of ≥20% with inhalation of <16 mg/mL methacholine. Diagnosis of PIBO was made according to the persistent respiratory symptoms (≥2 months), such as tachypnea, wheezing and rales, with unsatisfactory response to inhaled corticosteroids or bronchodilators unlike asthma, as well as the compatible findings of chest CT scan.

We excluded patients under 2 years because these patients underwent infant pulmonary function tests using multiple breath washout tests. Patients with a history of malignancy or organ transplantation, as well as those with active pneumonia with lobar consolidation during their chest CT scan, were also excluded.

### Chest CT scan

Chest CT scans were performed with one of five CT systems (Sensation 16, Sensation 64, Somatom Definition AS+, and Somatom Definition Flash from Siemens Medical Solutions, Erlangen, Germany; and Discovery CT 750 HD and Revolution CT from GE Healthcare, Waukesha, WI, USA). Tube voltages ranging from 70 to 140 kVp were chosen based on the patient’s weight. The automatic dose modulation technique was applied, if possible. Each patient was moved into a supine position, encouraged to inhale deeply, and to hold their breath during the examination, if possible. For children younger than eight years, sedation was performed under the supervision of pediatric anesthesiologists, and scanning was done without breath-holding.

All volume CT dose indices (CTDIvol) and dose length product values were recorded, from which we calculated CTDIvol in terms of size-specific dose estimates (SSDEs) with reference to American Association of Physicists in Medicine report 204 [15]. Effective radiation doses were estimated by multiplying the dose length product by age-specific conversion factors [16]. We also recorded the use of iodine contrast.

### Quantitative analyses of chest CT

CT scans were quantitatively analyzed by one radiologist, who was blinded to PFT results and clinical course, using dedicated imaging analysis software (Intellispace Portal, version 7.0, Philips Medical Systems, Cleveland, OH, USA). Both airway and air-trapping parameters were determined following the protocols outlined below.

#### Airway measurement

**Fig 1:**
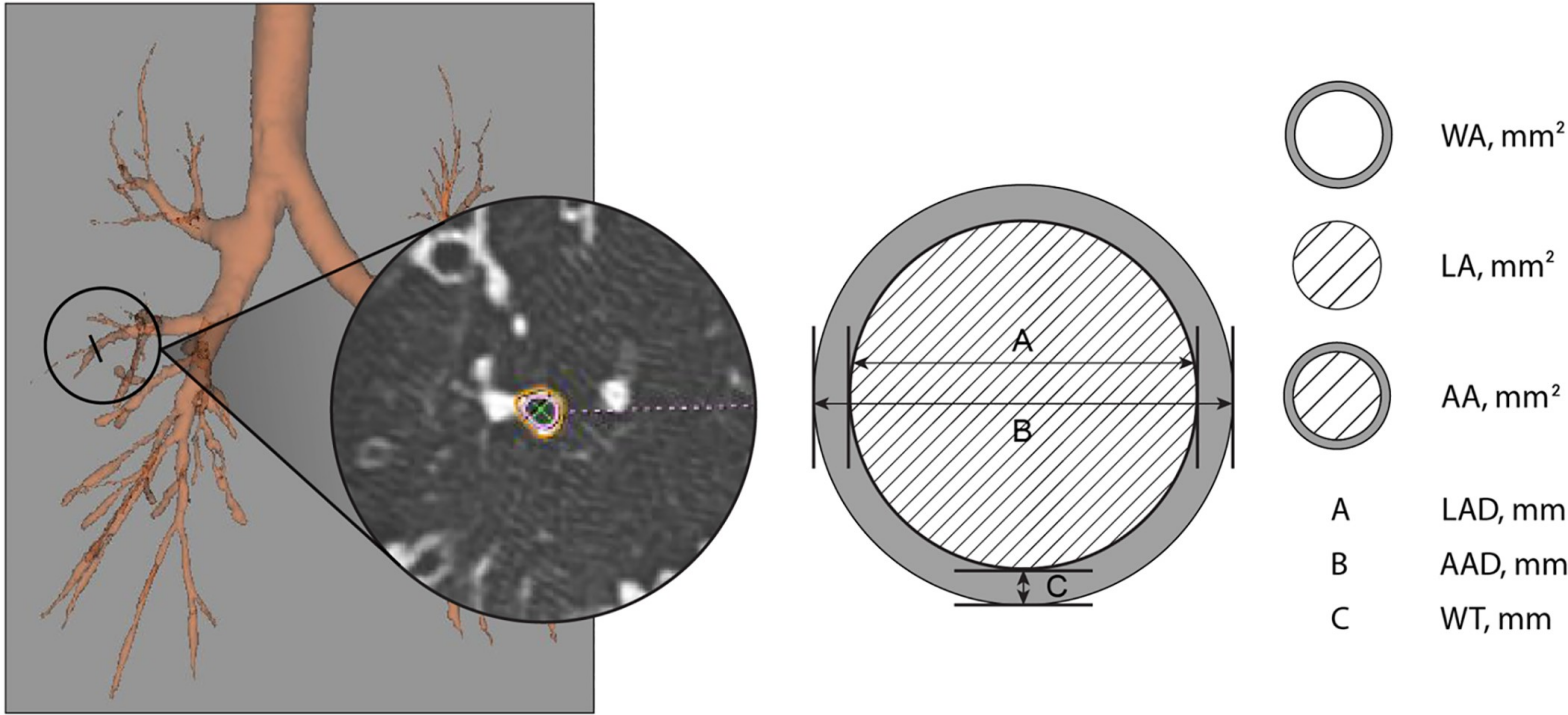
Schematic drawing of airway parameters. WA, wall area; LA, lumen area; AA, airway area; LAD, lumen average diameter; AAD, airway average diameter; WT, wall thickness.

To include the most distal branches, automatic segmentation of the airways was done first, followed by manual modification. After segmentation, a representative branch from each segment was selected. Specifically, left segments 1+2 and segments 7+8 were each counted as a single segment. As such, a maximum of 18 segmental and subsegmental bronchi could be identified per patient. The start and end points of each segmental and subsegmental bronchus were marked by the radiologist on either volume-rendered or cross-sectional images. We subsequently measured and calculated the following for each segmental and subsegmental bronchus: wall thickness (WT), wall area (WA), lumen average diameter, lumen area (LA), WA/LA ratio, airway average diameter, and airway area (Fig 1). For simplification, we grouped all segmental and subsegmental bronchi according to the pulmonary lobe in which they were physically located, and the mean value for each parameter was calculated across all bronchi within each lobe, as follows: right segments 1–3 (the right upper lobe, or RUL), right segments 4 and 5 (the right middle lobe, or RML), right segments 6–10 (the right lower lobe, or RLL), left segments 1–5 (the left upper lobe, or LUL), and left segments 6–10 (the left lower lobe, or LLL).

#### Air-trapping assessment

Measurements of air-trapping were performed using the previously published individualized threshold protocol [14]. Two small regions of interest (ROIs) were identified on the axial images from each chest CT in the areas most representative of normal and air-trapping parenchyma. The lung’s dependent area was avoided because its lung attenuation value could be inappropriately exaggerated. The mean CT attenuation values for each defined ROI were measured, and the average of the two attenuation values was used as the threshold to distinguish air-trapping areas from normal lung parenchyma. We defined air-trapping volume (ATV) of the whole lung or lobar lung as the lung volume when the attenuation level was below the established threshold. Each patient’s total lung volume was also measured, and the air-trapping ratio (ATR) was calculated between ATV and total lung volume. All measurements were also performed on the pulmonary lobes.

### Analysis of PFT

We included PFTs that were completed within one month of the associated chest CT scan. All tests were performed according to the American Thoracic Society (ATS) criteria [17]. Parameters of spirometry were measured with a computerized spirometer (Viasys Healthcare, Inc., Conshohocken, PA), calibrated according to age, sex, height, weight, and race [18], and expressed as the z-value. Estimates were obtained using the Global Lungs Initiative 2012 [19]. Forced vital capacity (FVC), FEV_1_, peak expiratory flow, and forced expiratory flow at 25–75% of the pulmonary volume (FEF_25-75_) were measured before and after bronchodilator inhalation. The impulse oscillometry (IOS) study measured the following parameters before and after bronchodilator inhalation: respiratory resistance, respiratory reactance, and reactance area at a frequency ranging from 5 to 20 Hz. The Z-score for IOS was obtained using the formula devised by Dencker et al. [20].

### Statistical analyses

Statistical analyses were completed using IBM SPSS Statistics for Windows, Version 22.0 (IBM Corp. Armonk, NY, USA). The Kolmogorov-Smirnov test was used to assess the normality of each dataset, and the correlations between CT measurements and PFT results were assessed using either the Pearson or Spearman correlation, depending on the normality of the variables. To analyze the airway parameters, we included the mean value for each segmental bronchus found in each lung (right and left) and lobe (RUL, RML, RLL, LUL, and LLL). The same process was used to assess the same set of airway parameters in the subsegmental bronchi. For air-trapping parameters, either the Pearson or Spearman correlation analysis was used as outlined above. P-values less than 0.05 were defined as statistically significant. To compensate for the multiple comparison problem, Bonferroni correction was applied to the airway parameters that correlated significantly with the PFT results. The significant p-value for those parameters was redetermined after the correction.

## Results

### Patient characteristics, PFT results and CT radiation dose

This study included 23 patients who received a total of 23 CT scans and 23 PFTs. They all were diagnosed with PIBO by conventional CT. This study population comprised 10 boys and 13 girls, with a mean age at the time of CT scan of 7.0 ± 3.3 years (ranging from 4 to 15 years and median of 6 years). There were five children aged four, six children aged five, and three children aged six. The ranges for height, weight, and body mass index were 99–169 cm, 14–67 kg, and 13.6–24.0 kg/m^2^, respectively.

The patients’ clinical characteristics are presented in Table 1. The most common symptom or sign was cough, followed by wheezing, dyspnea, and crackles. Co-morbidities were found in 12 patients (10 with asthma and two with bronchiectasis). None of the patients had severe clinical symptoms or required home oxygen.

**Table 1:** Patients’ demographics.

**Clinical characteristics**			
Signs and symptoms	n (%)	Ever confirmed causative infection	n (%)
Cough	17 (73.9)	*Mycoplasma pneumoniae*	6 (26.1)
Crackles	5 (21.7)	Influenza	2 (8.7)
Wheezing	11 (47.8)	RSV	1 (4.3)
Dyspnea	6 (26.1)	Adenovirus	1 (4.3)
Co-morbidities			
Asthma	10 (43.5)	Bronchiectasis	2 (8.7)
**Pulmonary function test**			
Spirometry	Z-score	Impulse oscillometry	Z-score
FVC	-1.549 ± 2.351	R5	1.685 ± 1.183
FEV_1_	-2.180 ± 2.391	R10	1.320 ± 0.902
FEV_1_/FVC	-1.556 ± 1.680	R20	0.863 ± 1.011
FEF_25-75_	-1.869 ± 1.909	X5	-2.704 ± 2.055
Change in FEV_1_ after BD, %		3.2 (0.75–17.25)	

Values are presented as mean ± standard deviation, median (interquartile range) or number (%)

RSV, respiratory syncytial virus; FVC, forced vital capacity; FEV_1_, forced expiration volume in one second; FEF_25-75_, forced expiratory flow at 25–75% of vital capacity; R5, resistance at 5 Hz; R10, resistance at 10 Hz; R20, resistance at 20 Hz; X5, reactance at 5 H; BD, bronchodilator

All 23 PFTs were deemed acceptable and reproducible according to the ATS criteria. The mean time interval (± standard deviation) between each CT scan and its associated PFT was 6.6 ± 8.5 days (ranging from 0–33 days). The spirometry parameters including FVC, FEV_1_, and FEF_25-75_ were reduced, and both R5 and R10 were increased while X5 was reduced in IOS study. Four (17.4%) patients had bronchodilator response in FEV_1_ (Table 1).

CT scans used the following tube voltages: two scans at 70 kVp, 12 scans at 80 kVp, six scans at 100 kVp, two scans at 120 kVp, and one scan at 140 kVp (S1 Table). Fifteen of the 23 CT scans also included an intravenous contrast injection. SSDE was determined to be 4.8 ± 5.8 mGy (range, 0.7–22.1 mGy), the dose length product was 166.7 ± 233.2 mGy-cm (range, 20.6–804.8 mGy-cm), and the effective dose was 2.3 ± 3.0 mSv (range, 0.4–10.5 mSv). All CT scans were of adequate quality for reading and analysis.

### Quantitative CT scan analyses

**Fig 2:**
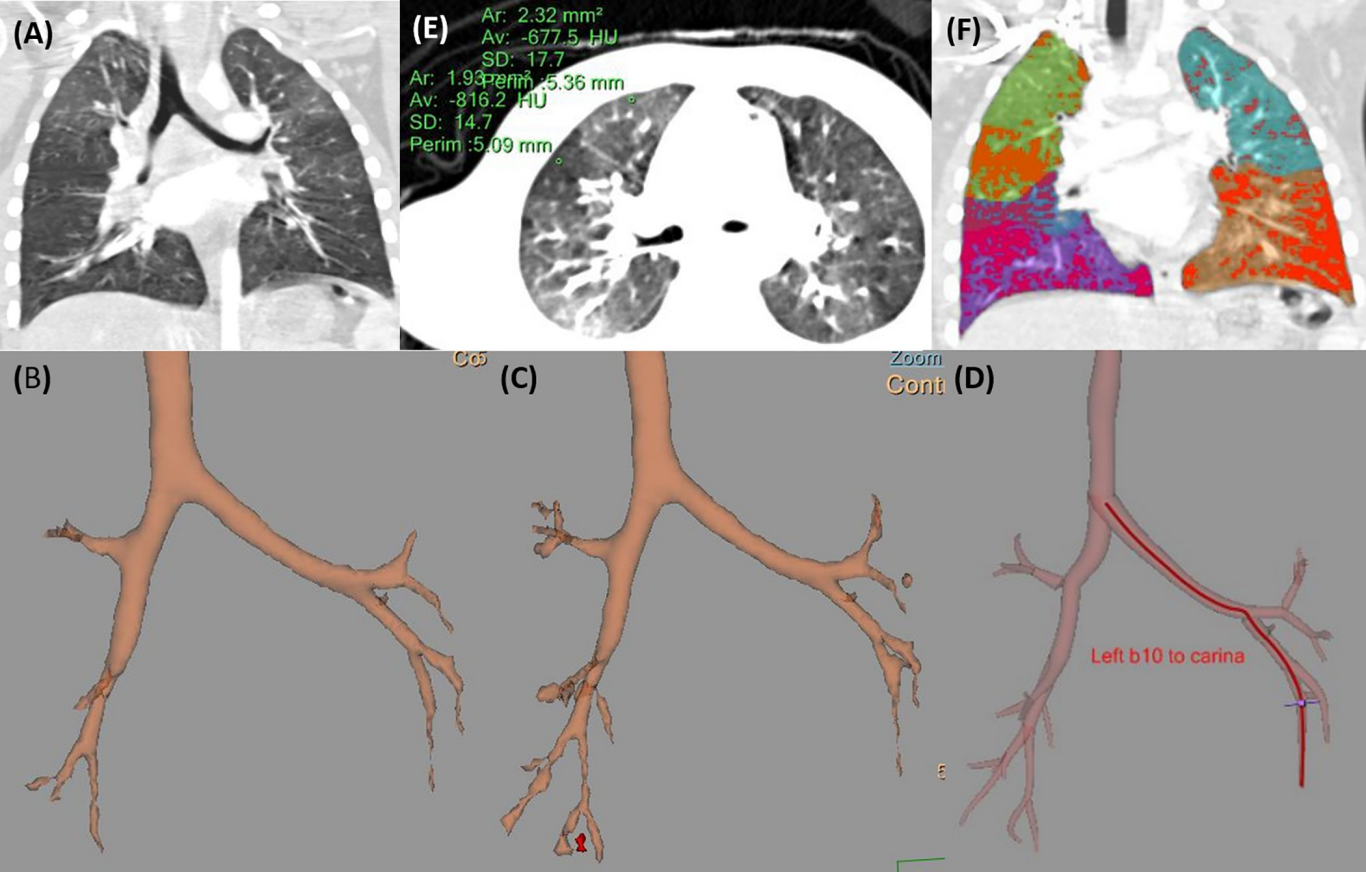
A representative case of successful quantitative analysis of a four-year-old girl with post-infectious bronchiolitis obliterans. (A) The coronal reconstructed CT image demonstrates bronchial wall thickening with mosaic attenuation in bilateral lungs. (B and C) Automatic airway segmentation (B) and subsequent manual editing (C) reveal all segmental bronchi in bilateral lungs. (D) Each segment is labeled. (E) Small regions of interest are drawn at normal and obviously air-trapping lung parenchyma to calculate the individualized threshold value of air-trapping. (F) Lung parenchyma area with CT attenuation value lower than the calculated threshold is marked with orange.

**Fig 3:**
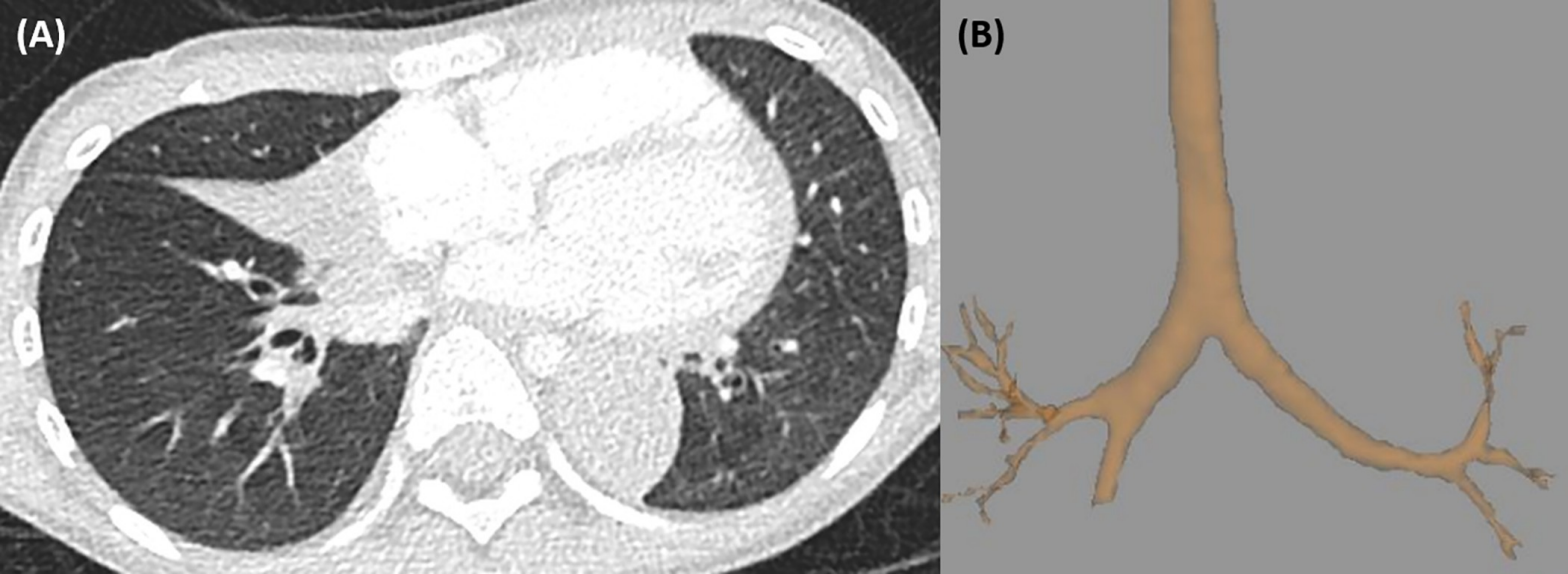
A representative case showing failure of an attempted quantitative airway analysis of a four-year-old boy with post-infectious bronchiolitis obliterans. (A) An axial CT image shows atelectasis of right middle lobe and left lower lobe. Airways are not delineable in the affected areas. (B) Airway segmentation failed in the corresponding and distal regions.

All relevant airway measurements for each pulmonary lobe and the air-trapping assessment results are summarized in Table 2. Airway measurements were successfully completed for a total of 371 of 414 segmental bronchi (89.6%) and 242 of 414 subsegmental bronchi (58.5%). The proportions of segmental bronchi analyzed for each lobe region were as follows: 66 (66/69, 95.7%) for the RUL, 39 (39/46, 84.8%) for the RML, 99 (99/115, 86.1%) for the RLL, 84 (84/92, 91.3%) for the LUL, and 83 (83/92, 90.2%) for the LLL. Of the five youngest patients (each 4 years of age), we successfully analyzed all segmental bronchi in three patients (Fig 2). Failed airway segmentation among the entire subjects were caused by atelectasis (n = 4, Fig 3), bronchiectasis (n = 1), mucus impaction (n = 1), and a motion artifact (n = 1).

**Table 2:** Quantitative CT airway and air-trapping parameters measured in 23 post-infectious bronchiolitis obliterans patients.

	Airway parameters							Air-trapping parameters	
	WT (mm)	WA (mm^2^)	LAD (mm)	LA (mm^2^)	WA/LA ratio (%)	AAD (mm)	AA (mm^2^)	ATV (cc)	ATR (%)
**Right lung**	1.4 ± 0.3	20.2 ± 5.8	2.9 ± 0.8	7.4 ± 4.1	363.7 ± 171.6	5.7 ± 0.9	27.7 ± 8.3	195.5 ± 323.5	22.1 ± 16.8
**RUL**	1.4 ± 0.3	20.4 ± 6.5	2.9 ± 0.8	7.4 ± 4.1	368.1 ± 186.2	5.8 ± 1.0	27.8 ± 8.7	63.4 ± 121.8	18.1 ± 14.6
**RML**	1.4 ± 0.3	20.0 ± 7.3	2.9 ± 1.2	7.8 ± 6.9	371.5 ± 200.3	5.7 ± 1.3	27.8 ± 12.6	41.2 ± 39.1	35.7 ± 20.0
**RLL**	1.4 ± 0.3	19.3 ± 6.0	2.8 ± 0.5	6.6 ± 2.4	355.3 ± 151.6	5.6 ± 0.8	25.9 ± 7.2	91.0 ± 170.3	20.9 ± 19.8
**Left lung**	1.3 ± 0.4	18.6 ± 7.5	2.8 ± 0.8	7.4 ± 4.7	336.9 ± 180.8	5.5 ± 1.1	26.0 ± 10.3	196.1 ± 311.8	26.3 ± 18.9
**LUL**	1.3 ± 0.4	16.9 ± 7.2	2.6 ± 0.9	6.4 ± 4.8	362.3 ± 227.7	5.2 ± 1.1	23.3 ± 10.0	84.2 ± 127.3	23.7 ± 17.1
**LLL**	1.4 ± 0.4	21.3 ± 8.9	3.1 ± 0.9	8.6 ± 5.4	323.7 ± 153.9	5.9 ± 1.2	29.9 ± 12.0	111.8 ± 193.7	27.6 ± 23.2

Data are mean ± standard deviation.

RUL, right upper lobe; RML, right middle lobe; RLL, right lower lobe; LUL, left upper lobe; LLL, left lower lobe; WT, wall thickness; WA, wall area; LAD, lumen average diameter; LA, lumen area; AAD, airway average diameter; AA, airway area; ATV, air-trapping volume; ATR, air-trapping ratio

Air-trapping assessments were successfully performed during all 23 CT scans. The mean attenuation value was -760 ± 92 HU in normal lung parenchyma and -851 ± 67 HU in obviously air-trapping lung parenchyma. The threshold for defining air-trapping, therefore, was established at -806 ± 75 HU.

### Correlations between airway CT parameters and PFT results

**Fig 4:**
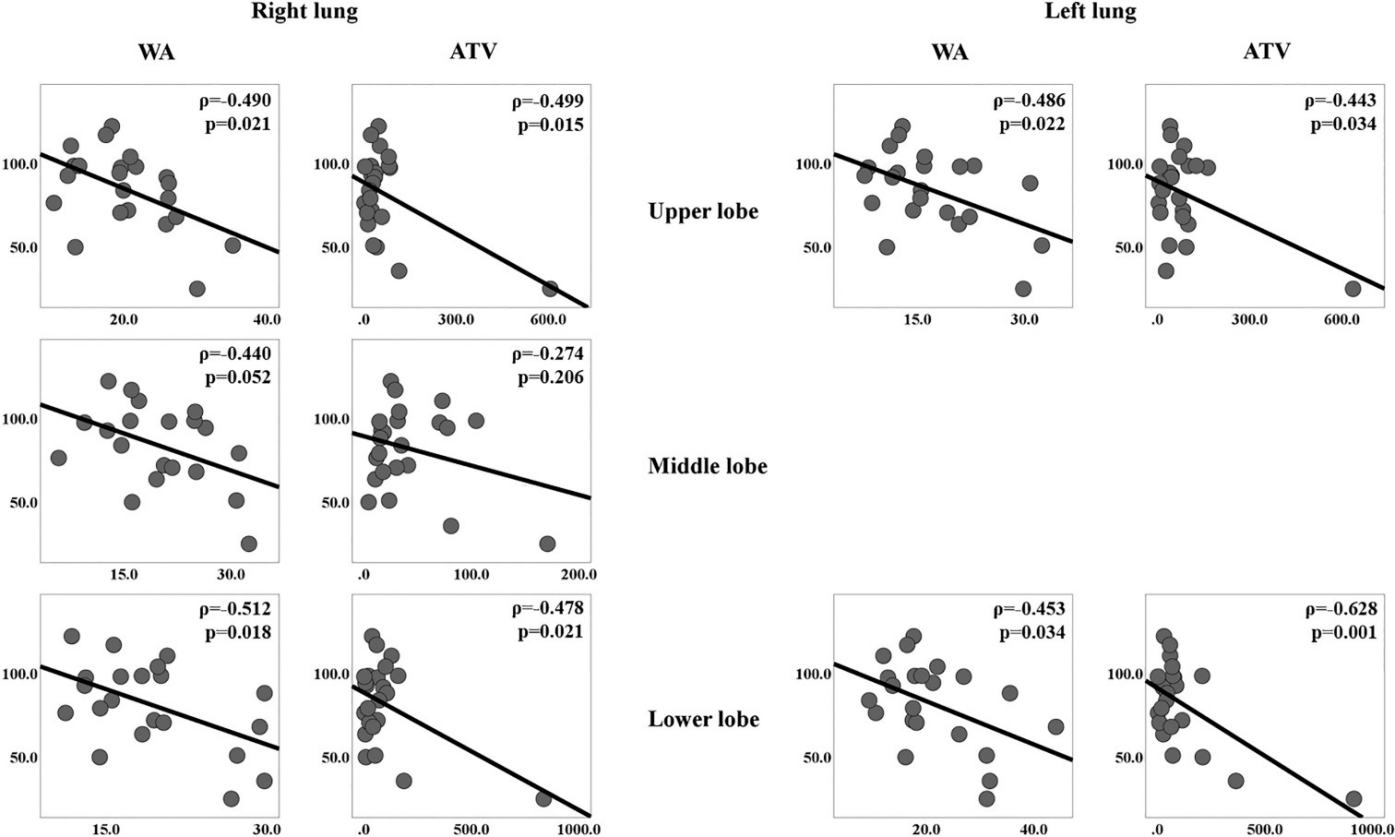
Scatter plots for each lobe of both lungs. Scatter plots demonstrating the correlation of wall area (WA) or air-trapping volume (ATV) with pre-bronchodilator forced expiration volume in one second (FEV1) in each pulmonary lobe of both lungs.

Although we found that the precise results from our correlation tests varied slightly between pulmonary lobes in each patient, we nonetheless found that WA exhibited a significant negative correlation with pre-bronchodilator FEV_1_ in both lungs (ρ = -0.481, p = 0.024 in the right lung and ρ = -0.452, p = 0.034 in the left lung) (Table 3). When the parameters were grouped by pulmonary lobe, we found that WT and WA exhibited correlations in most lobes (Table 3). Therefore, Bonferroni correction was applied for those two parameters, and the significant p-value was determined to be 0.025 (0.05/2). After correction, WA showed significant correlations with FEV_1_ in the RUL, RLL, and LUL (Fig 4). The IOS study, however, revealed no significant correlation with any airway parameters in any of the pulmonary lobes.

**Table 3:** Correlations between airway parameters and pre-bronchodilator FEV_1_.

	Airway parameters													
	WT		WA		LAD		LA		WA/LA ratio		AAD		AA	
	ρ	p-value	ρ	p-value	ρ	p-value	ρ	p-value	ρ	p-value	ρ	p-value	ρ	p-value
**Right lung**	-0.456	0.033	-0.481	0.024*	-0.048	0.831	-0.044	0.845	-0.195	0.384	-0.336	0.127	-0.357	0.102
**RUL**	-0.414	0.055	-0.490	0.021*	-0.101	0.656	-0.067	0.765	-0.041	0.858	-0.379	0.082	-0.396	0.068
**RML**	-0.486	0.030	-0.440	0.052	-0.012	0.959	0.000	0.999	-0.270	0.250	-0.264	0.261	-0.255	0.278
**RLL**	-0.462	0.035	-0.512	0.018*	-0.172	0.457	-0.192	0.405	-0.156	0.501	-0.449	0.041	-0.490	0.024
**Left lung**	-0.384	0.078	-0.452	0.034	-0.156	0.488	-0.217	0.332	-0.226	0.312	-0.389	0.073	-0.432	0.045
**LUL**	-0.430	0.046	-0.486	0.022*	-0.056	0.804	-0.089	0.692	-0.283	0.202	-0.350	0.110	-0.394	0.069
**LLL**	-0.384	0.078	-0.453	0.034	-0.235	0.292	-0.295	0.183	-0.173	0.443	-0.437	0.042	-0.471	0.027

* Statistically significant values after applying Bonferroni correction for WT and WA (p<0.025)

RUL, right upper lobe; RML, right middle lobe; RLL, right lower lobe; LUL, left upper lobe; LLL, left lower lobe; WT, wall thickness; WA, wall area; LAD, lumen average diameter; LA, lumen area; AAD, airway average diameter; AA, airway area

In the subsegmental bronchi, we identified a similar correlation between airway parameters and PFT, with WA demonstrating a significant negative correlation with pre-bronchodilator FEV_1_ in both lungs (ρ = -0.632, p = 0.006 in right lung and ρ = -0.577, p = 0.015 in left lung).

### Correlations between air-trapping CT parameters and PFT results

Of the two air-trapping-related CT parameters, only ATV was found to have a significant negative correlation with pre- and post-bronchodilator FEV_1_, as well as a positive correlation with respiratory reactance at 5Hz in the majority of pulmonary lobes (the RML was the exception). We found the relevant correlation coefficients (ρ) between ATV and pre- and post-bronchodilator FEV_1_ to be -0.472 (p = 0.023) and -0.474 (p = 0.022), respectively, in the right lung and -0.571 (p = 0.004) and -0.571 (p = 0.004) in the left lung (Table 4). The correlations between ATV and pre-bronchodilator FEV_1_ in each lobe are shown in Fig 4. The ρ between ATV and respiratory reactance at 5Hz was 0.693 (p < 0.001) in the RUL, 0.338 (p = 0.114) in the RML, 0.648 (p = 0.001) in the RLL, 0.644 (p = 0.001) in the LUL, and 0.723 (p < 0.001) in the LLL.

**Table 4:** Correlations between air-trapping parameters and pre-bronchodilator FEV_1_.

	Air-trapping parameters			
	ATV		ATR	
	ρ	p-value	ρ	p-value
**Right lung**	-0.472	0.023*	-0.219	0.315
**RUL**	-0.499	0.015*	-0.327	0.128
**RML**	-0.274	0.206	0.158	0.473
**RLL**	-0.478	0.021*	-0.273	0.207
**Left lung**	-0.571	0.004*	-0.396	0.061
**LUL**	-0.443	0.034*	-0.071	0.747
**LLL**	-0.628	0.001*	-0.478	0.021*

* Statistically significant values (p<0.05)

RUL, right upper lobe; RML, right middle lobe; RLL, right lower lobe; LUL, left upper lobe; LLL, left lower lobe; ATV, air-trapping volume; ATR, air-trapping ratio

## Discussion

It is not easy to obtain reliable PFT results from pediatric patients, even though it is important to assess their pulmonary function and monitor it periodically. In this study, we investigated the feasibility of using CT-based quantitative airway and air-trapping measurements to evaluate PIBO in children (considering the histopathology of PIBO as concentric narrowing and obliteration of small airways). The success rate of segmental bronchi measurement was almost 90%. Moreover, both airway measurements (WA) and air-trapping assessments (ATV) correlated with pulmonary function parameters, suggesting the possibility of using chest CT for quantitative evaluation and disease severity assessment in children with PIBO.

In this study, we demonstrated that it is technically feasible to obtain quantitative measurements of segmental airways using CT scans in children, even in preschoolers, and that this application holds potential clinical value for assessing PIBO patients. For adult patients with a history of smoking or COPD, quantitative airway analyses of CT scans have been widely used [21,22]. By contrast, no previous study of children has quantitatively analyzed segmental airways based on CT data, though two semi-quantitative analyses in children used scoring system based on qualitative image findings [5,13]. Our study is thus the first to evaluate quantitative chest CT assessment, including airway measurements, in children, and we demonstrated its potential clinical value for assessing PIBO patients.

In the present study, CT-based quantitative airway measurements were successfully completed for 89.6% of all segmental bronchi across all patients, suggesting that a reasonable degree of quantitative segmental bronchi analysis can be completed in children. The success rate for subsegmental bronchi, however, was only 58.5%, suggesting that these methods are not as effective at resolving particularly small bronchial regions while maintaining a low radiation dose. We argue, however, that even with the reduced spatial resolution of a pediatric chest CT, these results are sufficient to support their clinical application.

There were notable findings in the PFT results. The study population showed a low Z-score of FEF_25-75_ and a high Z-score of resistance at a low frequency, suggesting that this population has peripheral airway obstruction, which is compatible with PIBO. The parameters of spirometry and IOS showed reduced lung function and increased peripheral airway resistance even after bronchodilator inhalation (data not shown), which represented persistent airflow limitation in PIBO.

In assessing the correlations between CT-based airway measurements and PFT results, we identified a significant negative correlation between WA and FEV_1_ in the majority of the pulmonary lobes. FEV_1_ is known to be the most reproducible parameter that reflects the severity of obstructive lung disease, and its value decreases under conditions of increasing pathological symptoms. The WA widening can be from the inflammation surrounding the airway lumen as a feature of PIBO [3]. However, not only wall thickening, but also luminal narrowing and bronchiectasis can affect the results of WA, WT and LA, so all these parameters should be interpreted together. Moreover, we found that the degree of correlation varied depending on the lobe being examined, and we found no correlation between WA, ATV, and FEV_1_ in the RML. PIBO can produce a highly heterogeneous distribution of severity across the lung regions, suggesting that the variable degrees of correlation between CT-based measurements and PFT results for each lobe likely reflect the true heterogeneity of the disease.

Quantitative chest CT analysis also includes air-trapping assessment, and several studies have examined its clinical utility for adult patients. There are two critical differences between adults and children that should be taken into account when measuring ATV. First, the degree of CT attenuation in normal lungs changes depending on age in children [23]; second, respiratory control is often difficult to realize for children.

In this study, we applied the individualized threshold method originally established by Kim et al. [14] to measure ATV in PIBO patients, and our analyses identified a significant negative correlation between ATV and FEV_1_ in both lungs and in the majority of lobes, which is consistent with our WA measurement results. This finding is consistent with the known PIBO pathophysiology, which states that the greater is the trapped air volume, the greater is the severity of the disease. We expected that ATV measurements would have practical advantages because they require less time and effort than a complete airway analysis. We found that ATV also correlated with an IOS parameter (reactance at 5 Hz). Parameters measured by IOS have been shown to reflect the small airways in the lung periphery [24]. For example, previous work reported that impedance, resistance, and reactance at 5 Hz are unusually high in PIBO patients [25]. Overall, our results are consistent with this previous study, and together they suggest that ATV could reflect the true condition of the small airways. Regardless of the use of intravenous contrast, air-trapping was evaluated using the individualized threshold in this study and, as with previous study, significant results were obtained. However, further studies are needed on how contrast medium affects the evaluation of air-trapping in children.

The present study does have some limitations. First, we examined only 23 patients because we required a short amount of time between the CT and PFT to increase the validity of the correlation analysis. Second, this was a retrospective study that examined previously collected data, making it vulnerable to potential selection bias and to inconsistent CT scan protocols that could affect spatial resolution or alter the possibility of airway segmentation. Intravenous contrast use, contrast phase, and image reconstruction kernels can affect the bronchial wall thickness measurements, as demonstrated in a study with an adult population [26]. However, no one has studied those effects in children. The respiratory phase was not fixed, which can affect the attenuation value of the lung parenchyma, as well as other airway parameters, but that is unfortunately unavoidable when performing CT scans on children. Third, this study lacks longitudinal follow-up data. PFT does not precisely indicate disease severity, and as such, identifying a measurement that correlates well with the clinical outcome would provide a better demonstration of the benefit of using quantitative analysis in these patients. Fourth, neither inter-observer nor intra-observer reliability of the quantitative measurements could be calculated because only one radiologist performed each analysis once. Despite those limitations, this study provides meaningful evidence that quantitative CT analysis of pediatric PIBO patients can be leveraged for more effective diagnosis and treatment assessments. Even though these CT measurements can be a time-consuming procedure, selecting assessments such as WA and ATV could shorten the measuring time and make clinical applications more feasible. We suggest that future research be undertaken with a larger sample size and the measurement of long-term clinical outcomes.

## Conclusions

We found that effective quantitative airway measurements and air-trapping assessments based on chest CT of young children are technically feasible, and that some of their metrics correlate with pulmonary function parameters in pediatric PIBO patients, specifically WA measurements in the airway and ATV in air-trapping. Our findings suggest potential clinical applications of quantitative CT to estimate disease severity in children with PIBO whose PFT cannot be assessed due to limited compliance.

## Supporting information

S1 Table Patients information, CT acquisition data and radiation dose. (DOCX)
