# Supplementary material for: Quantitative CT and pulmonary function in children with post-infectious bronchiolitis obliterans
Source: PLoS One. 2019 Apr 1;14(4):e0214647. doi: 10.1371/journal.pone.0214647 (PMC6443232; doi:10.1371/journal.pone.0214647)
Supplement: S1 Table — (DOCX) [file pone.0214647.s001.docx]

**S1 Table. Patients information, CT acquisition data and radiation dose.**

| **Patient number** | **Year** | **Age** | **Gender** | **Height (cm)** | **Weight (kg)** | **CT scanner** | **kVp** | **contrast (Y/N)** | **SSDE (mGy)** | **DLP (mGy-cm)** | **effective dose (mSv)** |
| --- | --- | --- | --- | --- | --- | --- | --- | --- | --- | --- | --- |
| 1 | 2010 | 4 | female | 102 | 15 | Sensation 64 | 80 | Y | 5.2 | 147.7 | 2.7 |
| 2 | 2011 | 4 | male | 99 | 14 | Discovery CT 750 HD | 80 | N | 2.4 | 63.6 | 1.1 |
| 3 | 2012 | 4 | female | 108 | 22 | Somatom Definition AS+ | 100 | N | 2.9 | 74.8 | 1.3 |
| 4 | 2015 | 4 | male | 107 | 17 | Somatom Definition Flash | 70 | Y | 1.2 | 31.8 | 0.6 |
| 5 | 2016 | 4 | male | 106 | 17 | Somatom Definition Flash | 70 | N | 1 | 26.2 | 0.5 |
| 6 | 2011 | 5 | male | 112 | 17 | Sensation 64 | 80 | Y | 5.2 | 151.5 | 2.7 |
| 7 | 2012 | 5 | male | 124 | 29 | Discovery CT 750 HD | 100 | N | 2.9 | 69.2 | 1.2 |
| 8 | 2013 | 5 | male | 116 | 20 | Discovery CT 750 HD | 80 | N | 2.9 | 82.3 | 1.5 |
| 9 | 2014 | 5 | male | 113 | 18.5 | Somatom Definition AS+ | 80 | Y | 0.7 | 20.6 | 0.4 |
| 10 | 2015 | 5 | female | 103 | 15 | Revolution CT | 100 | Y | 1.2 | 33.7 | 0.6 |
| 11 | 2016 | 5 | female | 113 | 21 | Revolution CT | 100 | N | 1.3 | 35.5 | 0.6 |
| 12 | 2010 | 6 | female | 117 | 24 | Sensation 64 | 80 | Y | 5.6 | 183.6 | 2.4 |
| 13 | 2013 | 6 | female | 120 | 24 | Somatom Definition Flash | 100 | N | 1.8 | 52.2 | 0.7 |
| 14 | 2016 | 6 | female | 117 | 26 | Revolution CT | 80 | Y | 1.4 | 48.6 | 0.6 |
| 15 | 2016 | 7 | female | 128 | 23 | Revolution CT | 80 | Y | 1.4 | 41.4 | 0.5 |
| 16 | 2016 | 8 | female | 139 | 30 | Revolution CT | 80 | N | 1.4 | 46.8 | 0.6 |
| 17 | 2016 | 8 | female | 128 | 27 | Revolution CT | 80 | Y | 1.8 | 54 | 0.7 |
| 18 | 2010 | 9 | male | 132 | 40 | Discovery CT 750 HD | 140 | Y | 22.1 | 751.7 | 9.8 |
| 19 | 2008 | 10 | female | 143 | 35 | Sensation 64 | 80 | Y | 4.9 | 163.7 | 2.1 |
| 20 | 2009 | 11 | male | 141 | 33 | Sensation 64 | 80 | Y | 5.6 | 179.8 | 2.3 |
| 21 | 2011 | 12 | female | 152 | 50 | Somatom Definition Flash | 100 | Y | 3.4 | 110.8 | 1.4 |
| 22 | 2011 | 14 | female | 169 | 50 | Somatom Definition Flash | 120 | Y | 15.8 | 660 | 8.6 |
| 23 | 2008 | 15 | male | 167 | 67 | Sensation 16 | 120 | Y | 17.7 | 804.8 | 10.5 |
